# Acupuncture for Alcohol Use Disorder: A Meta-Analysis

**DOI:** 10.1155/2017/7823278

**Published:** 2017-01-12

**Authors:** Na Young Shin, Young Jin Lim, Chae Ha Yang, Cheongtag Kim

**Affiliations:** ^1^Department of Psychology, College of Social Sciences, Catholic University of Daegu, Gyeongsan-si, Republic of Korea; ^2^Department of Psychology, College of Social Sciences, Daegu University, Gyeongsan-si, Republic of Korea; ^3^Department of Physiology, College of Oriental Medicine, Daegu Haany University, Daegu, Republic of Korea; ^4^Department of Psychology, College of Social Sciences, Seoul National University, Seoul, Republic of Korea

## Abstract

Empirical research has produced mixed results regarding the effects of acupuncture on the treatment of alcohol use disorder in humans. Few studies have provided a comprehensive review or a systematic overview of the magnitude of the treatment effect of acupuncture on alcoholism. This study investigated the effects of acupuncture on alcohol-related symptoms and behaviors in patients with this disorder. The PubMed database was searched until 23 August 2016, and reference lists from review studies were also reviewed. Seventeen studies were identified for a full-text inspection, and seven (243 patients) of these met our inclusion criteria. The outcomes assessed at the last posttreatment point and any available follow-up data were extracted from each of the studies. Our meta-analysis demonstrated that an acupuncture intervention had a stronger effect on reducing alcohol-related symptoms and behaviors than did the control intervention (*g* = 0.67). A beneficial but weak effect of acupuncture treatment was also found in the follow-up data (*g* = 0.29). Although our analysis showed a significant difference between acupuncture and the control intervention in patients with alcohol use disorder, this meta-analysis is limited by the small number of studies included. Thus, a larger cohort study is required to provide a firm conclusion.

## 1. Introduction

Alcohol use disorder is characterized by maladaptive patterns of alcohol consumption, which lead to health and social problems [1]. Excessive alcohol consumption is an important risk factor for various medical diseases, such as cancers and cardiovascular disease [1], and can cause alcohol-related liver disease and nutritional deficiencies [2]. Moreover, some evidence suggests that sustained alcohol abuse may result in neural abnormalities [3] and brain damage [2]. Individuals suffering from alcoholism can experience impaired neurocognitive functioning, including difficulties with executive functions and working memory [4], and dysfunction in several brain regions, including the white matter, cerebellum, frontal cortex, hypothalamus, and hippocampus [2, 4]. The harmful consequences associated with alcohol abuse and the high prevalence of alcohol use disorder [5] require an effective intervention in a healthcare setting. Epidemiological data indicate that patients with alcohol use disorder are largely untreated despite the efficacy of treatment, which may reflect a need for treatment alternatives.

Acupuncture emerged as a treatment for addiction in the 1970s, and the first report, from a Hong Kong study, indicated that it is a relatively safe complementary and alternative medical approach [6]. Since then, several acupuncture treatment protocols have been established to treat substance abuse. A five-point auricular protocol was developed by the National Acupuncture Detoxification Association in the 1980s, and a body point electrical stimulation protocol was designed by a Chinese doctor in the 1990s [7]. Researchers have attempted to elucidate the therapeutic effects of acupuncture on reducing addiction-related symptoms and regulating brain dysfunction using various treatment protocols [8, 9]. Despite methodological differences in acupuncture points, treatment durations, and types of control conditions among studies [9], a recent meta-analysis study demonstrated a significant beneficial effect of acupuncture on addiction-related clinical symptoms, such as withdrawal/craving and anxiety, with a medium to large effect size, although the data were heterogeneous and had a significant publication bias [8].

A substantial number of studies have suggested the efficacy of acupuncture interventions to ameliorate alcohol-related behaviors [10–13] and modify the relevant brain systems of animals [13–16]. These studies indicate that acupuncture stimulation at various acupoints, such as* Zusanli* (ST36) and* Shenmen* (HT7), reduces alcohol consumption [10, 16, 17] and modulates the reward circuit in the brain [15, 16, 18] of rats. However, results have been inconsistent in humans. Some randomized controlled studies have reported that the effect of acupuncture on reducing clinical symptoms related to alcohol addiction is significantly stronger than that of control conditions [19–23], whereas other studies found a comparable effect [24–28]. Due to lack of treatment guidelines specific to alcohol use disorder, these studies adopted different study designs and treatment protocols, which may have contributed to the conflicting results. Although two studies reviewed previous findings on the effect of acupuncture on alcoholism, no study has provided a general overview of this issue. Furthermore, no study has offered a systematic overview of the magnitude of the effect of acupuncture interventions for this disorder. Thus, this study integrated available data to examine the effects of acupuncture treatment on patients with alcohol use disorder.

## 2. Methods

### 2.1. Search Strategy and Study Selection

Potentially relevant articles were identified through a PubMed literature search using the keywords [acupuncture OR electroacupuncture OR “acupoint stimulation” OR transcutaneous OR electrostimulation AND alcohol] for articles published until 23 August 2016. Two researchers (Na Young Shin and Young Jin Lim) searched the articles independently. The reference lists of review articles were also inspected. The inclusion criteria were the following: (1) being published in a peer-reviewed English-language journal, (2) use of randomized controlled trials (RCTs), (3) assessing the effects of acupuncture on psychological variables in individuals with a primary alcohol problem, and (4) reporting statistics that could be converted to effect sizes.

### 2.2. Data Extraction

The same two authors (Na Young Shin and Young Jin Lim) cross-checked the recorded variables, including year of publication, type of intervention, treatment protocol, sample size for the last intervention, mean and standard deviation of age, and psychological assessment results (mean and SD or *t*, *F*, and *p* statistics). When psychological outcomes were assessed at several time points after the treatment, the results from the last intervention with a sample size >10 were extracted to examine the effect size of the treatment. Additionally, follow-up data were recorded to examine any long-term effect of treatment.

### 2.3. Statistical Analyses

The statistical analysis was conducted using Comprehensive Meta-Analysis ver. 3 software (Biostat Inc., Englewood, NJ, USA). Hedges' *g* was calculated to estimate the effect sizes of acupuncture on psychological variables, such as alcohol craving, alcohol use, and anxiety. Hedges' *g* represents the difference in the means for the acupuncture and control treatment conditions divided by the pooled SD and corrected for biases due to small sample size [29]. Positive effect sizes indicate positive effects of the acupuncture treatment compared with that of the control condition. Cochran's *Q* statistic [30] and *I*^2^ index were used to test the heterogeneity of the effect sizes across studies. When *p* value for the *Q* statistic was less than 0.1, the data were considered heterogeneous. A random-effects model was applied to obtain an average effect size, since the number of studies included in the meta-analysis was small. Publication bias was also examined by visually inspecting funnel plots and using Egger's regression intercept test [31]. We performed subsequent analyses on data from at least three studies for each psychological variable.

## 3. Results

### 3.1. Study Characteristics

**Figure 1:**
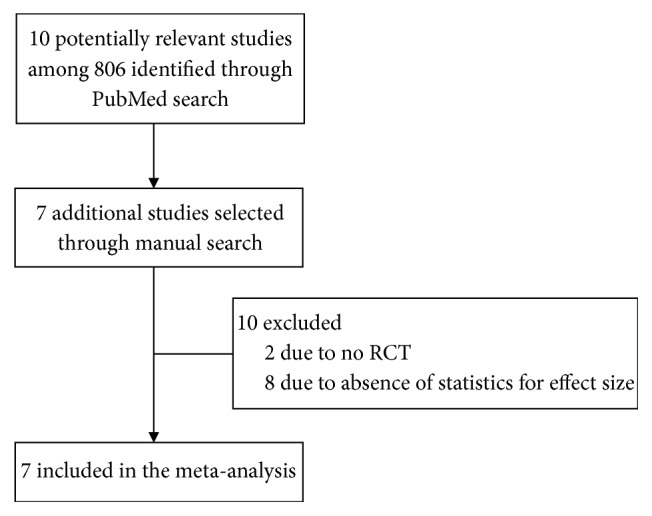
Search strategy used to select the studies in the meta-analysis.

In total, 806 articles were identified via the PubMed search, and 10 relevant articles were selected for further full-text analysis after a review of titles and abstracts. An additional seven studies were selected for full-text inspection via a manual search of the reference list from the reviewed studies. Based on the inclusion criteria, two studies were excluded because they were not RCTs, and eight were excluded due to the lack of statistics required to calculate effect sizes (Figure 1). The final seven studies were included in the meta-analysis to examine the effect sizes of acupuncture treatment on psychological variables. In total, 243 patients with alcohol use disorder (mean age [SD] 44.1 [4.2] years) were included in the analyses. Of these, 129 (mean age [SD] 43.6 [3.4] years) received acupuncture treatment and 114 were treated under the control condition (mean age [SD] 44.6 [5.2] years).

We rereviewed 7 studies selected via a PubMed search and 10 selected through a manual search of reference lists for the follow-up data. Among these articles, three reported statistics appropriate for conversion to effect sizes for the follow-up data. Two studies reported data 6 months after treatment, and one study reported data 12 months after treatment. In these three studies, 297 patients with alcohol use disorder (144 and 153 in acupuncture and control groups, resp.) participated in a follow-up survey.

The studies were conducted in the United States (*n* = 3), Switzerland (*n* = 1), Germany (*n* = 1), Ireland (*n* = 1), and England (*n* = 1). The treatment protocols varied regarding the acupoint location, type of control condition, and treatment duration (Table 1). Five studies treated patients by inserting a needle into several acupoints in each ear. Two studies stimulated body points with or without ear stimulation. Four studies treated control patients with a placebo needle or under a completely different type of intervention, such as relaxation or transdermal stimulation, whereas the remaining studies inserted needles into nonspecific points. The patients were treated for 2 weeks to 3 months, and the treatment duration per session was 15–45 min.

### 3.2. Effects of Acupuncture on Psychological Variables

**Figure 2:**
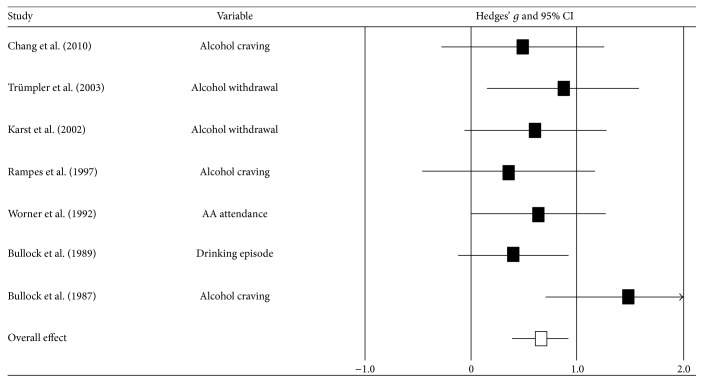
Overall effect size of the psychological variables in each study.

To estimate the overall effect of acupuncture on psychological variables, studies reporting alcohol-related symptoms or behaviors, such as alcohol craving, alcohol withdrawal, and the number of drinking episodes, were synthesized in the meta-analysis. As shown in Table 2 and Figure 2, the acupuncture intervention had a more positive effect on alcohol-related symptoms and behaviors than did the control conditions, in which effect size was medium (*g* = 0.67). A significant effect of acupuncture on alcohol craving was detected for each psychological variable, with a medium to large effect size (*g* = 0.78). Additionally, the difference between acupuncture and control interventions showed a medium to large effect size of 0.78 (*p* = 0.065) for alcohol use behaviors. However, no difference was found between the acupuncture and control conditions with regard to anxiety (*g* = 0.32).

A significant difference was observed between the acupuncture and control conditions, with a small effect size for the follow-up data (*g* = 0.29), indicating a positive but relatively weak long-term effect of acupuncture treatment.

### 3.3. Heterogeneity and Publication Bias

In meta-analysis, heterogeneity and publication bias may lead to erroneous conclusions. The former occurs when study outcomes vary between studies due to methodological and clinical differences, and the latter occurs when studies with statistically significant results are more likely to be published than those with nonsignificant results.

The *Q* test revealed no significant heterogeneity for the overall effect (*Q* = 6.39, *I*^2^ = 6.17, and *p* = 0.38), anxiety (*Q* = 1.23, *I*^2^ < 0.01, and *p* = 0.54), or the follow-up data (*Q* = 1.77, *I*^2^ < 0.01, and *p* = 0.41), but significant heterogeneity was detected for alcohol craving (*Q* = 4.76, *I*^2^ = 57.99, and *p* = 0.09) and alcohol use (*Q* = 6.90, *I*^2^ = 71.03, and *p* = 0.03). No subgroup analysis could be performed for the heterogeneous variables due to the small sample size of the study. Visual inspection of funnel plots and Egger's test revealed no significant publication bias for any of the psychological variables (intercept = 2.70, *t* = 1.05, and *p* = 0.34 for overall effect; intercept = −20.37, *t* = 0.43, and *p* = 0.74 for alcohol craving; intercept = 2.72, *t* = 0.48, and *p* = 0.72 for anxiety; intercept = 1.09, *t* = 0.20, and *p* = 0.88 for alcohol use; intercept = −1.27, *t* = 0.91, and *p* = 0.53 for the follow-up data).

## 4. Discussion

The present study is the first meta-analysis to examine the effect of acupuncture treatment on patients with alcohol use disorder and to provide data on the magnitude of this effect on alcohol-related clinical symptoms and behaviors. Our analysis shows that acupuncture treatment had a stronger effect on reducing specific clinical symptoms, including alcohol craving/withdrawal, and on modulating alcohol-related behaviors compared to a control intervention, with effect sizes of 0.6 to 0.8. Given that these results were drawn from data collected at the last posttreatment visit after completing 2 weeks to 3 months of treatment, the significant results may reflect the effect of multiple trials rather than that of a single trial. The magnitude of the long-term effect of acupuncture on alcohol-related symptoms and behaviors was statistically significant but weak, with a small effect size. Notably, this meta-analysis was limited by the small number of studies; thus, the findings should be interpreted cautiously. Well-controlled large cohort studies are needed to elucidate whether acupuncture is effective for treating alcohol use disorder over short-term and long-term periods.

Although the sample size of the present study was insufficient to allow a reliable interpretation of the meta-analytic findings, the magnitude of the effect of acupuncture treatment was relatively strong, with no publication bias and low heterogeneity across studies. The treatment protocols of the studies included in our meta-analysis varied, particularly regarding acupoint location, type of control condition, and treatment duration. Despite several methodological differences, the studies indicated a positive effect of acupuncture treatment on alcohol-related symptoms and behaviors. Our results are partly consistent with findings of a recent meta-analysis that observed a significant difference between acupuncture and control conditions in terms of reducing postintervention withdrawal/craving and anxiety in patients with a substance use disorder [8]. Interestingly, this prior meta-analysis also showed that acupuncture had unique effects on some variables on alcohol use disorder such as treatment dropout. That study also reported that traditional Chinese medicine acupuncture had a better effect than auricular acupuncture for withdrawal/craving symptoms. Given that most studies reviewed in our study applied auricular acupuncture, additional studies are needed to clarify which type of acupuncture is more effective for alcoholism.

In conclusion, this meta-analysis suggests that acupuncture treatment has a more significant effect than control interventions on the alcohol-related symptoms and behaviors of patients with alcohol use disorder. However, as the number of studies included in the meta-analysis was small, further RCTs are required to yield a definitive conclusion about the effect. Furthermore, additional studies are needed to explore acupuncture treatment protocols for alcohol use disorder and to elucidate the long-term effects of such interventions on larger samples.

## Figures and Tables

**Table 1:** Summary of studies included in the meta-analysis.

Study	Acupuncture/control		Treatment protocol			Assessment	F/U
	*N*	Age^*∗*^	Intervention	Acupoint	Treatment periods		
Chang et al. (2010)	17/10	46/51	Needle/relaxation	Ear	Twice weekly for 10 wks	Alcohol craving^#^	
						Anxiety	
Trümpler et al. (2003)	15/16	45/49	Needle/sham laser	Ear	Daily until the end of withdrawal	Alcohol	
						withdrawal^#^	
Karst et al. (2002)	17/17	46/41	Needle/placebo needle	Ear & body	Daily for 2 wks	Alcohol	
						withdrawal^#^	
						Anxiety	
Bullock et al. (2002)^*∗∗*^	98/115	NR	Needle, specific/nonspecific	Ear	Daily for 3 wks	Alcohol craving	12 mon later
Rampes et al. (1997)	10/12	39/40	Electroacupuncture, specific/nonspecific	Ear	Once weekly for 6 wks	Alcohol craving^#^	6 mon later
						Anxiety	
						Alcohol use	
Worner et al. (1992)	19/21	42/42	Needle/sham transdermal	Body	Thrice weekly for 3 mon	AA attendance^#^	6 mon later
Bullock et al. (1989)	32/25	NR	Needle, specific/nonspecific	Ear	Once weekly for the first 2 wks, thrice weekly for the next 4 weeks, and twice weekly for the last 2 wks	Drinking episode^#^	
						Alcohol use	
Bullock et al. (1987)	19/13	NR	Needle, specific/nonspecific	Ear	Daily for 5 days, thrice weekly for the next 4 weeks, and twice weekly for 45 days	Alcohol craving^#^	
						Alcohol use	

^*∗*^Mean of age of all participants in the study.

^*∗∗*^Included only for an investigation of long-term effect.

^#^Psychological variables included in the analysis for the overall effect size estimation.

F/U, included in the follow-up data analysis; mon, month; NR, not reported; specific/nonspecific, specific point or nonspecific point to addiction; wks, weeks.

**Table 2:** Effects of acupuncture on psychological variables in alcoholics.

Psychological variable	Studies, *N*	Acupuncture, *N*	Control, *N*	ES	95% CI	*z*	*p*
Overall effect	7	129	114	0.656	0.389, 0.920	4.837	<0.001
Alcohol craving	3	46	35	0.781	0.080, 1.481	2.184	0.029
Anxiety	3	42	45	0.320	−0.110, 0.750,	1.459	0.145
Alcohol use	3	61	50	0.777	−0.048, 1.602,	1.847	0.065
Long-term effect	3	144	153	0.286	0.059, 0.514	2.463	0.014

CI, confidence interval.
